# Trends and Outcome from Radical Therapy for Primary Non-Metastatic Prostate Cancer in a UK Population

**DOI:** 10.1371/journal.pone.0119494

**Published:** 2015-03-05

**Authors:** David C. Greenberg, Artitaya Lophatananon, Karen A. Wright, Kenneth R. Muir, Vincent J. Gnanapragasam

**Affiliations:** 1 Public Health England, National Cancer Registration Service [Eastern Office], Cambridge, United Kingdom; 2 Division of Health Sciences, Warwick Medical School, University of Warwick, Warwick, United Kingdom; 3 Institute of Population Health, University of Manchester, Manchester, United Kingdom; 4 Academic Urology Group, Department of Surgery & Oncology, University of Cambridge, Cambridge, United Kingdom; 5 Translational Prostate Cancer Group, Hutchison/MRC Research centre, University of Cambridge, Cambridge, United Kingdom; Sun Yat-sen University, CHINA

## Abstract

**Background:**

Increasing proportions of men diagnosed with prostate cancer in the UK are presenting with non-metastatic disease. We investigated how treatment trends in this demographic have changed.

**Patient and Methods:**

Non-metastatic cancers diagnosed from 2000–2010 in the UK Anglian Cancer network stratified by age and risk group were analysed [n = 10,365]. Radiotherapy [RT] and prostatectomy [RP] cancer specific survival [CSS] were further compared [n = 4755].

**Results:**

Over the decade we observed a fall in uptake of primary androgen deprivation therapy but a rise in conservative management [CM] and radical therapy [p<0.0001]. CM in particular has become the primary management for low-risk disease by the decade end [p<0.0001]. In high-risk disease however both RP and RT uptake increased significantly but in an age dependent manner [p<0.0001]. Principally, increased RP in younger men and increased RT in men ≥ 70y. In multivariate analysis of radically treated men both high-risk disease [HR 8.0 [2.9–22.2], p<0.0001] and use of RT [HR 1.9 [1.0–3.3], p = 0.024] were significant predictors of a poorer CSM. In age-stratified analysis however, the trend to benefit of RP over RT was seen only in younger men [≤ 60 years] with high-risk disease [p = 0.07]. The numbers needed to treat by RP instead of RT to save one cancer death was 19 for this group but 67 for the overall cohort.

**Conclusion:**

This study has identified significant shifts in non-metastatic prostate cancer management over the last decade. Low-risk disease is now primarily managed by CM while high-risk disease is increasingly treated radically. Treatment of high-risk younger men by RP is supported by evidence of better CSM but this benefit is not evident in older men.

## Introduction

The incidence of prostate cancer is rising in the UK and worldwide [1–2]. This rise however has not been associated with a significant change in cancer death rates. This raises the issue of how treatment can be tailored to address the complex heterogeneity of the disease and reduce morbidity from over-treatment. It has been shown that radical therapy of all cancers can result in significant over-treatment without conferring survival benefit [3]. Conversely, effective radical treatment can reduce disease progression and improve cancer specific survival [CSS] [4–5]. Another major shift in treatment is the increasing use of Radical Prostatectomy [RP], driven in part by the introduction of laparoscopic and robotic approaches [6]. RP has indeed been shown to be an effective treatment with many studies suggesting superiority over other radical options [7]. There remains however, significant uncertainty on the strength of the evidence for this benefit.

Many of these developments have only come to light towards the end of the last decade and the impact on therapy patterns in the UK have not to date been well studied. This is particularly relevant as the UK has been at the forefront of research in the use of active surveillance [AS] for low risk disease and increased radical treatment for more aggressive disease [5, 8–9]. In this report, we interrogated a well-annotated cancer registry database to investigate patterns of primary non-metastatic prostate cancer treatment over the last decade. Our primary interest was to determine if the uptake of radical therapy had altered over time and how these changes were influenced by disease and patient characteristics.

## Patients and Methods

### Patient cohort and data collation

Prostate cancers [ICD10 site: C61] diagnosed in the Anglia Cancer Network area between 2000 and 2010 and registered by the National Cancer Registration Service—Eastern Office [NCRS(E)] were interrogated for this study. Data elements recorded by NCRS[E] include non-identifiable patient demographics, TNM [fifth edition to 2009 and seventh edition in 2010] stage, Gleason grade, PSA and details of treatment administered. Recent reports have highlighted the completeness of staging information at NCRS[E] [10]. No personal information or patient identifiable data was obtained or used for this study at any stage. The data was analysed completely anonymously. As a result no formal ethics was necessary for this study. From this cohort we identified all men who presented with non—metastatic disease. Risk groups were ascribed based on the NICE guidelines criteria [2]. Electronic death notifications were received from the Office of National Statistics. Vital status was also checked using the Health and Social Care Information Centre Personal Demographics Service batch tracing system http://systems.hscic.gov.uk/demographics/pds/]. Cause of death was classified as prostate cancer specific when listed in Cause 1[a], 1[b], or 1[c] of the death certificate, except when a markedly worse prognosis cancer was listed in Cause 1[a]. Survival times were calculated from the date of diagnosis to date of death or date of censorship [30 September 2013]. The median follow up was 6.9 years.

### Treatments

We focused on 4 therapy modalities; Radical Prostatectomy [RP], External beam radiotherapy [RT], primary androgen deprivation therapy [PADT] and conservative management [CM]. The first documented mode of management within one year of diagnosis was assigned as the primary treatment. CM included men on both active surveillance and watchful waiting as the registry recorded both these treatment modalities as “Watch policy”. This was distinct from cases where there was missing data on treatments received. External beam radiotherapy within one year of diagnosis in cases with non-metastatic cancer [with or without androgen depletion] was considered as radical radiotherapy. Notably 88.2% of patients treated with primary radiotherapy also had concurrent androgen deprivation therapy though we were not able to determine the duration of ADT or radiotherapy dose.

### Statistical analysis

Annual incidence rates [IR] per 100,000 were calculated with each treatment for the whole group and then stratified by age and risk groups. Overall, age and risk group specific incidence curves were then plotted for visual trends. To compute incidence rates we summed cases and population between 2000–2005 and 2006–2010 and then calculated for five year rates. Difference in incidence rate ratio [IRR] between the two periods, the standard error and confidence interval for IRR were calculated using EpiBasic software [http://www.folkesundhed.au.dk/uddannelse/software]. To compare mortality rates between RP and RT, we computed the prostate cancer mortality rate difference [MRD] per 100,000 person-days [P-D] for each group and then stratified by age and risk group [EpiBasic software]. p<0.05 was considered statistically significant different for both analysis. Cox regression analyses was performed to build a predictive model for time-to-event data using R Commander plug-in EZR [Easy R] version 1.23 [1] and IBM SPSS 20. Test assumptions of proportional Hazard ratio [PH] by visual and rho chi-square were also performed and all the test results met the PH assumption. Finally, we calculated the Number Needed to Treat [NNT] as an indicator to measure the effectiveness of treatments with cancer specific mortality as the outcome [http://www.calctool.org/CALC/prof/medical/NNT].

## Results

### Therapy trends in primary non-metastatic prostate cancer

**Fig 1:**
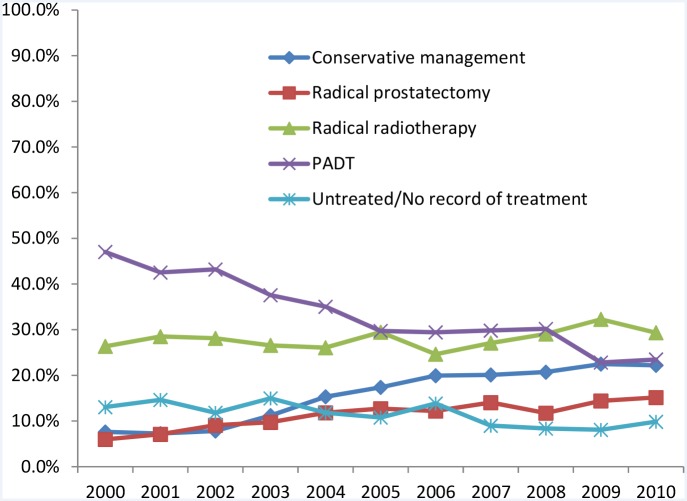
Proportions of men with non-metastatic cancer treated by different modalities in the Anglia Cancer Network from 2000–2010.

10, 787 men with non-metastatic local disease were identified for this study of whom 422 [3.9%] did not have a documented treatment. The final cohort therefore included 10,365 men; 1435 men treated by RP, 3320 by RT, 3590 by PADT and 2020 by CM [no active treatment]. Over the decade there was a more than 50% reduction in the use of PADT as a treatment modality [p<0.0001] [Table 1] [Fig. 1]. In contrast, the use of RP had more than doubled [p<0.0001]. The use of non- active treatment or CM as primary management had also tripled from 7% to 22% in the same interval [p<0.0001]. RT usage however remained relatively stable over the decade.

**Table 1:** Incidence rate changes in prostate cancer treatment modality for non-metastatic disease between 2000–2005 and 2006–20.

Treatment	Percentage 2000–2005	Percentage 2006–2010	SE	Z	p-value	Trend
**Conservative management**	14%	23%	0.00792	-11.651	0.000	Increase
**Radical prostatectomy**	12%	15%	0.0069	-4.631	0.000	Increase
**Radical radiotherapy**	32%	32%	0.00932	-0.037	0.970	No Change
**PADT**	42%	30%	0.00951	13.098	0.000	Decrease

### Radical therapy uptake in risk stratified groups

**Fig 2:**
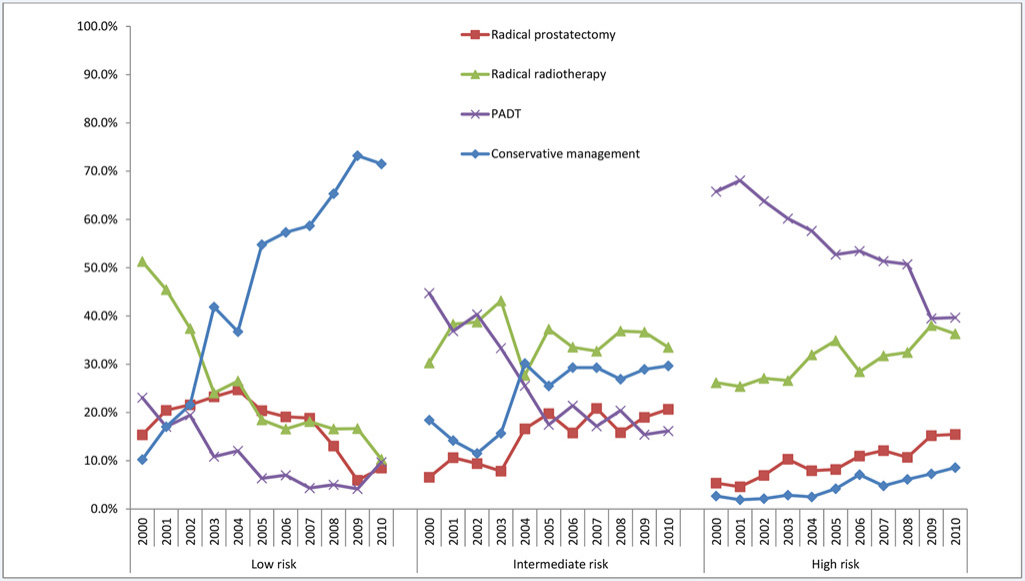
Proportions of men with non-metastatic cancer treated by different modalities and stratified by NICE risk group from 2000–2010.

The population was then sub-divided into risk groups based on NICE criteria to investigate the effect of risk group on therapy trends [Table 2] [Fig. 2]. In low-risk cancers the use of CM increased by five-fold over the decade, while uptake of RT and RP fell significantly [p < 0.0001 for both] [Table 2] [Fig. 2]. Intermediate-risk cancers also showed a similar trend with a 1.5–2 fold increase in the use of CM over the decade [p<0.0001]. Here however there was also an increase in the uptake of RP amounting to 17–18% of the cohort in the latter half of the decade [p<0.0001] [Fig. 2]. RT uptake however remained static in this group [Table 2]. In high-risk cancers the use of both RP and RT increased significantly [p<0.0001 for both]. RP uptake increased by 3-fold while RT uptake at a more modest rate [Fig. 2]. Use of PADT however fell across all groups regardless of risk category [Fig. 2].

**Table 2:** Incidence rate changes in prostate cancer treatment modality for non-metastatic disease stratified by NICE risk group between 2000–2005 and 2006–201.

	Treatment	Percentage 2000–2005	Percentage 2006–2010	SE	Z	p-value	Trend
**low risk**	**Conservative management**	35%	66%	0.0255	-12.048	0.000	Increase
	**Radical prostatectomy**	22%	13%	0.0192	4.720	0.000	Decrease
	**Radical radiotherapy**	30%	16%	0.0212	6.819	0.000	Decrease
	**PADT**	13%	6%	0.0149	4.830	0.000	Decrease
**Intermediate risk**	**Conservative management**	21%	29%	0.0150	-5.110	0.000	Increase
	**Radical prostatectomy**	14%	19%	0.0128	-3.979	0.000	Increase
	**Radical radiotherapy**	36%	35%	0.0163	0.718	0.473	No change
	**PADT**	29%	18%	0.0141	8.192	0.000	Decrease
**High risk**	**Conservative management**	3%	7%	0.0062	-6.386	0.000	Increase
	**Radical prostatectomy**	8%	13%	0.0088	-6.019	0.000	Increase
	**Radical radiotherapy**	30%	34%	0.0133	-3.194	0.001	Increase
	**PADT**	59%	46%	0.0141	9.530	0.000	Decrease

### Radical therapy uptake in risk stratified group subcategorised by age

We next asked how the combination of age and risk-group influenced treatment trends [Table 3]. Over the decade men ≤ 60y with low-risk disease were significantly more likely to be treated by CM rather than radical therapy [p<0.0001]. Men ≤ 60y with high-risk disease however were more likely to be treated radically with RP instead of RT as primary therapy [p = 0.009] [Table 3]. Management of intermediate-risk disease remained relatively unchanged. In men aged 60–69y, low and intermediate-risk disease were also more likely to be managed by CM towards the end of the decade [p<0.0001 and p<0.028 respectively]. Again men in this age group with high-risk disease were more likely to receive RP by the end of the decade [p< 0.0001] though the proportions treated by RT were unchanged. Amongst intermediate-risk men there was a trend to reduced use of RT but no change in the proportions of men treated by RP [Table 3]. In men ≥ 70y with low-risk disease CM use nearly doubled in the decade while rates of all other treatments fell [p<0.0001]. RP uptake increased in intermediate and high-risk cancers but represented small proportions of men treated [4%]. There was however a significant increase in men treated by RT for high-risk disease amounting to over a third of this cohort by the end of the decade [p<0.0001] [Table 3]. There were no major shifts in treatment trends in men ≥ 80 years and PADT remained the most common treatment for this demographic.

**Table 3:** Incidence rate changes in prostate cancer treatment modality for non-metastatic disease stratified by age and NICE risk group between 2000–2005 and 2006–2010.

Age	Risk	Treatment	Percentage 2000–2005	Percentage 2006–2010	SE	Z	p-value	Trend
**< 60 years**	**Low risk**	**Conservative management**	22.5%	53.4%	0.056	-5.717	0.000	Increase
		**Radical prostatectomy**	46.5%	26.1%	0.055	3.719	0.000	Decrease
		**Radical radiotherapy**	26.4%	16.1%	0.047	2.192	0.028	Decrease
		**PADT**	5.6%	4.3%	0.025	0.487	0.626	No change
	**Intermediate risk**	**Conservative management**	5.8%	13.3%	0.034	-2.194	0.028	Increase
		**Radical prostatectomy**	52.1%	55.1%	0.055	-0.560	0.575	No change
		**Radical radiotherapy**	33.9%	27.4%	0.050	1.300	0.194	No change
		**PADT**	8.3%	4.2%	0.025	1.634	0.102	No change
	**High risk**	**Conservative management**	1.3%	5.2%	0.020	-1.990	0.047	Increase
		**Radical prostatectomy**	31.4%	38.1%	0.050	-1.349	0.177	No change
		**Radical radiotherapy**	49.7%	37.2%	0.051	2.417	0.016	Decrease
		**PADT**	17.6%	19.5%	0.041	-0.451	0.652	No change
**60–69**	**Low risk**	**Conservative management**	28.2%	61.0%	0.037	-8.877	0.000	Increase
		**Radical prostatectomy**	25.5%	15.9%	0.030	3.212	0.001	Decrease
		**Radical radiotherapy**	37.3%	18.9%	0.033	5.577	0.000	Decrease
		**PADT**	9.1%	4.2%	0.018	2.679	0.007	Decrease
	**Intermediate risk**	**Conservative management**	13.8%	21.4%	0.022	-3.421	0.001	Increase
		**Radical prostatectomy**	23.8%	27.2%	0.025	-1.345	0.179	No change
		**Radical radiotherapy**	49.6%	42.7%	0.028	2.428	0.015	Decrease
		**PADT**	12.9%	8.8%	0.017	2.390	0.017	Decrease
	**High risk**	**Conservative management**	2.0%	4.4%	0.009	-2.567	0.010	Increase
		**Radical prostatectomy**	16.2%	25.9%	0.021	-4.630	0.000	Increase
		**Radical radiotherapy**	46.9%	47.7%	0.025	-0.307	0.759	No change
		**PADT**	34.9%	22.0%	0.023	5.690	0.000	Decrease
**70–79**	**low risk**	**Conservative management**	48.9%	79.0%	0.045	-6.692	0.000	Increase
		**Radical prostatectomy**	2.7%	0.0%	0.011	2.532	0.011	Decrease
		**Radical radiotherapy**	24.9%	11.6%	0.036	3.682	0.000	Decrease
		**PADT**	23.5%	9.4%	0.035	4.062	0.000	Decrease
	**Intermediate risk**	**Conservative management**	26.1%	33.9%	0.024	-3.294	0.001	Increase
		**Radical prostatectomy**	1.5%	4.5%	0.009	-3.252	0.001	Increase
		**Radical radiotherapy**	33.5%	35.5%	0.025	-0.808	0.419	No change
		**PADT**	38.9%	26.1%	0.024	5.389	0.000	Decrease
	**High risk**	**Conservative management**	2.0%	5.4%	0.008	-4.077	0.000	Increase
		**Radical prostatectomy**	1.6%	4.0%	0.007	-3.278	0.001	Increase
		**Radical radiotherapy**	26.9%	36.3%	0.020	-4.642	0.000	Increase
		**PADT**	69.5%	54.3%	0.021	7.209	0.000	Decrease
**>80+**	**low risk**	**Conservative management**	78.3%	86.7%	0.104	-0.808	0.419	NA
		**Radical prostatectomy**	0.0%	0.0%	0.000	N/A		NA
		**Radical radiotherapy**	0.0%	0.0%	0.000	N/A		NA
		**PADT**	21.7%	4.7%	0.065	2.622	0.009	NA
	**Intermediate risk**	**Conservative management**	36.2%	58.3%	0.053	-4.129	0.000	NA
		**Radical prostatectomy**	0.0%	0.0%	0.000	N/A		NA
		**Radical radiotherapy**	6.6%	5.3%	0.025	0.493	0.622	NA
		**PADT**	57.2%	36.4%	0.053	3.914	0.000	NA
	**High risk**	**Conservative management**	6.3%	14.8%	0.020	-4.186	0.000	NA
		**Radical prostatectomy**	0.0%	0.0%	0.000	N/A		NA
		**Radical radiotherapy**	5.3%	5.5%	0.015	-0.098	0.922	NA
		**PADT**	88.4%	79.7%	0.024	3.592	0.000	NA

### Comparative cancer mortality outcomes between radical therapies

**Fig 3:**
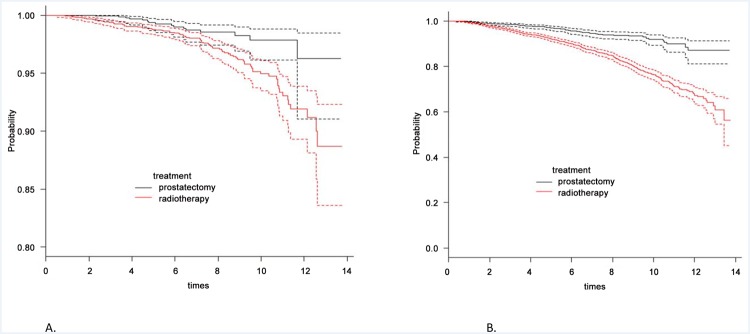
Cumulative (A) Cancer specific and (B) Overall survival from radical prostatectomy and radical radiotherapy. Dashed lines represent 95% confidence intervals.

**Fig 4:**
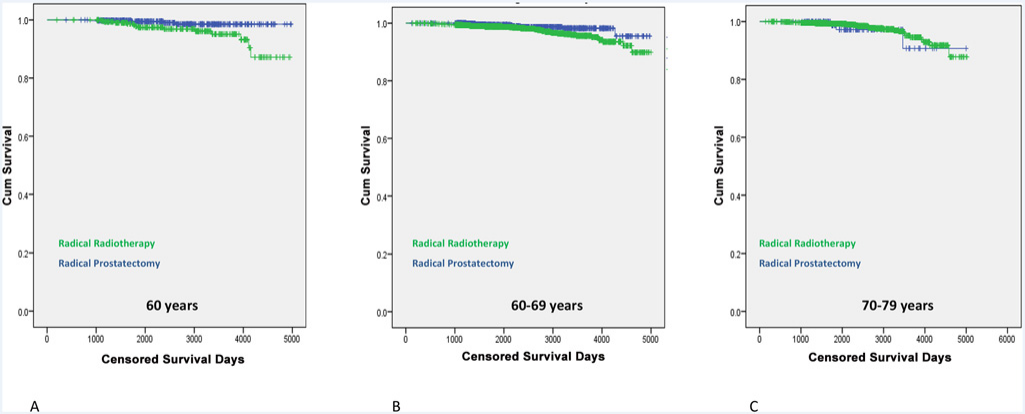
Cumulative cancer specific survival from radical prostatectomy and radical radiotherapy in age stratified groups.

This study has revealed increasing uptake of RP for intermediate and high-risk local disease. We asked if this trend was supported by evidence of better cancer-specific survival [CSS] outcomes compared to RT. Overall cancer mortality from radical therapy [1,435 RP and 3320 RT treated men] was 2.7%; 3.2% for RT treated men, and 1.7% for RP. Cumulative survival curves demonstrated overlap between the two groups in the first few years after treatment before beginning to diverge [Fig. 3A]. Men in the RT group however had worse overall survival rates suggesting significantly more co-morbidity in this group [Fig. 3B]. In multivariate analysis, predictors of a worse CSS outcome were presentation with high-risk disease or RT as the primary treatment [Table 4]. To define this better we further stratified the cohort by age and risk-group. This analysis demonstrated a trend to benefit from surgery but only in the high-risk younger men [<60y] [p = 0.07] [Table 5]. Age stratified survival plots further illustrated this point whereby men <60y had demonstrably better CSS rates after RP as compared to RT [Fig. 4A]. These benefits were less apparent in men aged 60–69y and non-existent in men>70y [Fig. 4B and 4C]. Finally, we performed a numbers needed to treat [NNT] analysis to quantify the RP benefit compared to RT. For the whole cohort the NNT by RP instead of RT to save one cancer death was 67 [Table 6]. This number fell to 19 in men ≤ 60y and with high-risk disease. For all other groups the NNT were in excess of 50 or favoured RT over RP.

**Table 4:** Multivariate analysis of variables predictive of cancer specific mortality from radical therapy.

Variable		Hazard Ratio	p value
**Age (y)**	60–69	0.8 (0.4–1.5)	0.5
	70–79	0.8 (0.4–1.5)	0.5
**Risk group**	Intermediate	2.8 (0.9–8.4)	0.05
	High	8.0 (2.9–22.2)	<0.0001
**Treatment**	Radiotherapy	1.9 (1.0–3.3)	0.024

**Table 5:** Comparative cancer specific mortality between radical prostatectomy and radical radiotherapy in groups stratified by age and risk type.

Age	Risk	Type of treatment	Prostate cancer specific mortality per 100000 P-D	MRD [Mortality rate difference per 100000]	SE	Approx. 95% CI		p-value
**< 60 years**	low	**Radical prostatectomy**	0.00	n/a	n/a	n/a	n/a	n/a
		**Radical radiotherapy**	0.00					
	intermediate	**Radical prostatectomy**	0.21	-0.16	0.43	-1.01	0.68	0.683
		**Radical radiotherapy**	0.38					
	high	**Radical prostatectomy**	0.94	-1.89	0.98	-3.81	0.03	0.070
		**Radical radiotherapy**	2.83					
**60–69 years**	low	**Radical prostatectomy**	0.00	-0.51	0.30	-1.09	0.07	0.136
		**Radical radiotherapy**	0.51					
	intermediate	**Radical prostatectomy**	0.12	-0.47	0.23	-0.93	-0.02	0.095
		**Radical radiotherapy**	0.60					
	high	**Radical prostatectomy**	1.03	-0.63	0.47	-1.56	0.29	0.224
		**Radical radiotherapy**	1.66					
**70–79 years**	low	**Radical prostatectomy**	4.20	4.2	4.2	-4.03	12.42	0.001
		**Radical radiotherapy**	0.00					
	intermediate	**Radical prostatectomy**	0.00	-0.79	0.25	-1.29	-0.3	0.357
		**Radical radiotherapy**	0.79					
	high	**Radical prostatectomy**	1.45	0.26	1.06	-1.82	2.34	0.790
		**Radical radiotherapy**	1.19					
**80+ years**	low	**Radical prostatectomy**	n/a	n/a	n/a	n/a	n/a	n/a
		**Radical radiotherapy**	n/a					
	intermediate	**Radical prostatectomy**	n/a	n/a	n/a	n/a	n/a	n/a
		**Radical radiotherapy**	2.16					
	high	**Radical prostatectomy**	n/a	n/a	n/a	n/a	n/a	n/a
		**Radical radiotherapy**	6.35					

**Table 6:** Data of the numbers needed to treat by radical prostatectomy versus radical radiotherapy to save 1 cancer specific mortality event.

	Age <60 years			Age 60–69 years			Age 70–79 years		
	low	intermediate	high	low	intermediate	high	low	intermediate	High
ARR	n/a	0.004	0.052	0.015	0.012	0.017	-0.167	0.0182	-0.0055
RRR	n/a	0.457	0.702	1	0.808	0.424	infinity	1	-0.2153
NNT	n/a	247	19	66	86	59	-6	55	-180

ARR- absolute risk reduction, RRR—relative risk reduction, NNT-numbers needed to treat.

## Discussion

The key findings of this study in non-metastatic cancers is a global fall in the use of PADT, increased radical therapy for intermediate and high-risk disease and the emergence of non-active treatment as the commonest option for low-risk cancer. Studies from other investigators have found both similar and different results. Dinan *et al*, 2011, in a US study reported a similar fall in PADT use in men aged ≥ 67y between 1999 to 2007 [11]. Concomitantly, the percentage of men receiving no active treatment increased by 50%. An Australian study of 2,774 men also reported increases in use of CM for low-risk cancer but only in older men [12]. Jacobs *et al*, 2013, looked at the use of new radical radiotherapy and surgical techniques in the SEER Cancer Registry [2004–2009] [13]. Here they found a two-fold increase in the use of these methods in men with low-risk cancer. This is in contrast to our present study which, while undertaken over a similar timeframe, actually revealed a fall in the use of radical therapy in low-risk men. Only one other UK study has explored treatment changes over time. Fairley *et al*, 2009, reported changes in therapy in the Northern and Yorkshire region from 2000–2006 [14]. This study also reported a fall in the use of PADT and increase use of RP but did not separate the outcomes by risk group. Our study is therefore the first to provide a risk-stratified analysis of treatment changes in a UK non-metastatic prostate cancer population.

In terms of radical therapy we found age and risk specific shifts for each modality. RT rates in intermediate and low-risk cancers were trending downwards over the decade but were increasing in men ≥ 70y with high-risk disease. The benefits of this change in practice is now supported by the 2009 and 2011 publications of the SPCG7 and MRC/PRO7 trials showing improved CSS by adding RT to PADT [4–5]. RP uptake, conversely, is rising in younger men with high-risk disease. This latter change in practice is again supported by recent data [albeit not randomised] from the EMPACT group and others showing the potential benefits of surgery in this demographic [15–16]. The absolute benefit of RP over RT however has been a point of controversy for some time [7, 17–18]. A number of studies have published comparisons but have been criticised for the lack of an optimal RT regime [17]. A significant strength of our cohort is that over 88% of RT patients received concurrent ADT though the radiotherapy dosage and ADT duration would have evolved over time. Within this context, RP appeared to show an overall CSS advantage. The main benefit however was in men ≤ 60y with high-risk disease which supports the change in practice observed in our cohort. In NNT analysis only this group demonstrated a reasonable treatment number to save one cancer death. To our knowledge this is the first UK study to compare RP and RT outcomes and report this finding. Our study does need longer follow up given the slow history of the disease but this in fact favours younger men who will have longer life expectancy. In this context the updated SPCG-4 randomised trial of RP versus watchful waiting is worth noting [19]. Over the 20 years of follow up the NNT by RRP has continued to fall and now stands at 8. Here again the main benefits have been seen in men ≤ 65y and with higher risk disease. This study did not include a RT arm and it would have been intriguing to assess the comparison after such a long follow up. Results from the ProtecT trial may be partly able to address this though most of the men recruited had low and intermediate disease [20–21]. The large NNT in our other subgroups suggest that RP confers at best marginal benefits over RT and this is unlikely to be significantly altered in longer follow up. This is particularly relevant when considering that RP has been shown to have worse immediate functional debility and impact on quality of life, compared to RT [22–23].

Our study clearly has a number of inherent limitations using as it does registry based information though the NCRS [East] is well-known for the accuracy and completeness of its data collection and collation. We acknowledge that this study only involves a single cancer network though there are no particular suspicions that our population is very different from the rest of the UK. As mentioned we could not differentiate between active surveillance and watchful waiting in men with no active treatment. Finally, our results comparing RP and RT outcomes is based on relatively short follow up and may become more pronounced as the follow up continues. We do however believe that our results are more representative of current clinical practice because of the high percentage of men on combination RT and ADT.

In conclusion, we report a significant shift in the management of non-metastatic prostate cancer over the last decade in a UK population. Within the limitations of a registry based study, these results suggest a move towards age and risk-appropriate treatment of non-metastatic prostate cancer. This is particularly reflected in the increasing use of non-active treatment for low-risk cancer. We further report an increasing use of RP for younger men with high-risk disease and demonstrate evidence suggesting a benefit in CSS. Overall these trends are very encouraging for the goal of tailored treatment for patients but will have implications for health resource allocation which will need to be considered by service providers.
